# MethyQA: a pipeline for bisulfite-treated methylation sequencing quality assessment

**DOI:** 10.1186/1471-2105-14-259

**Published:** 2013-08-23

**Authors:** Shuying Sun, Aaron Noviski, Xiaoqing Yu

**Affiliations:** 1 Department of Epidemiology and Biostatistics, Case Western Reserve University, Cleveland 44106, Ohio, USA; 2 Department of Mathematics, Texas State University, San Marcos 78666, Texas, USA; 3 Department of Electrical Engineering and Computer Sciences, Case Western Reserve University, Cleveland 44106, Ohio, USA

**Keywords:** DNA methylation, Next generation sequencing, Alignment, BRAT, Quality assessment

## Abstract

**Background:**

DNA methylation is an epigenetic event that adds a methyl-group to the 5’ cytosine. This epigenetic modification can significantly affect gene expression in both normal and diseased cells. Hence, it is important to study methylation signals at the single cytosine site level, which is now possible utilizing bisulfite conversion technique (i.e., converting unmethylated Cs to Us and then to Ts after PCR amplification) and next generation sequencing (NGS) technologies. Despite the advances of NGS technologies, certain quality issues remain. Some of the more prevalent quality issues involve low per-base sequencing quality at the 3’ end, PCR amplification bias, and bisulfite conversion rates. Therefore, it is important to conduct quality assessment before downstream analysis. To the best of our knowledge, no existing software packages can generally assess the quality of methylation sequencing data generated based on different bisulfite-treated protocols.

**Results:**

To conduct the quality assessment of bisulfite methylation sequencing data, we have developed a pipeline named MethyQA. MethyQA combines currently available open-source software packages with our own custom programs written in Perl and R. The pipeline can provide quality assessment results for tens of millions of reads in under an hour. The novelty of our pipeline lies in its examination of bisulfite conversion rates and of the DNA sequence structure of regions that have different conversion rates or coverage.

**Conclusions:**

MethyQA is a new software package that provides users with a unique insight into the methylation sequencing data they are researching. It allows the users to determine the quality of their data and better prepares them to address the research questions that lie ahead. Due to the speed and efficiency at which MethyQA operates, it will become an important tool for studies dealing with bisulfite methylation sequencing data.

## Background

In a mammalian genome, DNA methylation is an epigenetic event that involves the addition of a methyl-group (CH_3_) to 5’ cytosines following with guanines (i.e., C_p_G sites, where “p” stands for phosphate). This epigenetic modification plays an important role in cancerous cells. In fact, DNA methylation is one of the most common molecular changes in several cancers including breast, ovarian, and colon cancers [1-15]. DNA methylation can silence important tumor suppressor genes such as *p16, ER, and PR*[9]. It often occurs at the early stage of tumor development and can be easily detected in a small amount of DNA [16,17]. Thus it can be used as an early identifier in cancerous cells. Furthermore, its reversible characteristic, or demethylation (i.e., methylation can be removed), makes it a possible target for therapeutic demethylation drugs. For these reasons, identifying cancer methylation patterns has become an extremely important topic in the area of cancer epidemiology.

There are different types of cancer methylation patterns such as differential methylation and partial methylation, which play significant roles in tumor development and growth [18-20]. In order to identify these patterns, it is critically important to obtain methylation signals at the single C_p_G site level. With the bisulfite-treatment technique (i.e., converting unmethylated C to T) combined with advanced high throughput sequencing technologies, it is now possible to obtain methylation signals at the C_p_G site level. Over the last several years, a few leading research groups have successfully generated bisulfite-treated methylation sequencing data [21-27]. These data are extremely large. For example, the methylation sequencing data of one study may occupy gigabytes and even terabytes of hard-drive space depending on the coverage, size of sequencing regions, and number of samples.

There are different quality issues in giant sequencing data and it is challenging to preprocess and analyze such data. For example, in some experiments we see that 3’ end reads have dramatically low qualities, some have a lot of Ns at the 5’ and 3’ ends of sequencing reads, some k-mer sequences are unexpectedly highly represented, and some have a large number of duplicated reads. Although several tools have been successfully developed to align bisulfite-treated reads and call methylation signals [21-23,28-32], few packages have been developed for the quality assessment of bisulfite sequencing, except the recent SAAP-RRBS pipeline [33]. SAAP-RRBS consists of four modules including reads assessment and clean-up, alignment, C_p_G site methylation extraction, and annotation for C_p_G sites. This is a useful tool designed for the Reduced Representation Bisulfite Sequencing (RRBS) protocol [34], but not for whole genome sequencing or any other bisulfite-treated protocol. Although, in theory the workflow can be easily extended to analyze whole genome sequencing data, in practice it can be challenging due to the alignment speed. Furthermore, it does not have the feature of comparing the DNA sequence structure of different regions, as our new program will include. Therefore, there is still a need to develop a quality assessment tool for bisulfite-treated methylation sequencing data.

Bisulfite-treated DNA methylation sequencing has its own characteristics that may lead to different quality issues. For example, bisulfite treatment causes damage to DNA, resulting in fragmentation of long molecules [35]. Furthermore, bisuflite treatment may not be complete, and incomplete bisulfite conversion will affect methylation signal/ratio estimates. In addition, methylation in mammalian DNA generally occurs at C_p_G sites, which are often found in C_p_G islands that are regions with high GC contents and are likely to be repetitive regions. The high GC content and the repetitive regions tend to affect DNA sequencing, and after sequencing the distribution of A, C, G, and T in a bisulfite-treated genome (or target regions) is dramatically shifted because unmethylated C is converted to T. Any or all of these factors may affect the sequencing quality and results. It is critical to develop an efficient quality assessment package for bisulfite sequencing data generated based on different protocols to assist the accurate identification of methylation patterns. To meet this urgent need, we have developed a pipeline that incorporates both the currently available quality assessment programs and our new program with novel features.

## Implementation

### The workflow of our pipeline

**Figure 1:**
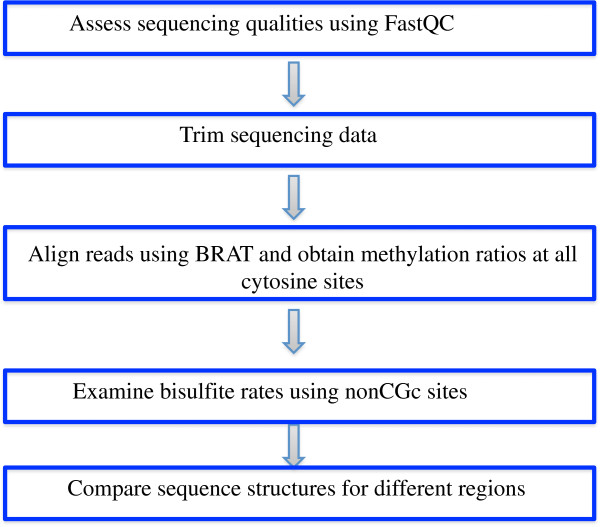
The workflow of our pipeline MethyQA.

The workflow of our pipeline (see Figure 1) is explained below wherein Steps 4 and 5 are our new features.

Step 1: Assess sequencing qualities using FastQC [36]. FastQC assesses sequencing qualities, sequence content, GC content, per base N content, duplication levels and so on. Though FastQC is not designed for bisulfite-treated methylation sequencing data (for example, it cannot assess bisulfite conversion rates), it still produces very informative diagnostic plots.

Step 2: Trim sequencing data. Quite often, sequencing quality is very low at the 3’ end in Illumina data. Low quality untrimmed reads will not be aligned. It is necessary to include a trimming step and trimming off low quality reads can improve NGS alignment result [37]. In our pipeline, two trimming options are provided: dynamic trimming (i.e., trimming based on quality scores using the trim function of the BRAT package [30]) and trimming off a fixed number of bases. In addition, adaptor trimming [38,39] is also included as an option.

Step 3: Align sequencing data using BRAT and obtain methylation ratios at all cytosine sites. After trimming, BRAT [30] is utilized as a default alignment tool. After alignment, the pipeline generates the methylation ratio file using the ACGT-count function of the BRAT package. The output files are methylation ratios of all cytosines on both forward and reverse strands for each sample.

As for the choice of the alignment tool in Step 3, we choose BRAT as the default. BRAT is an efficient tool for mapping FASTQ format short-reads by building a hash table for the reference genome. It is a very user-friendly software package and produces comparable results. Compared to other alignment tools [29,31,32,40-43], BRAT has several superior features. First, it uses relatively less memory [43]. Second, it does not limit read length. Third, it can align both single-end and paired-end reads. Fourth, it can account for overlapping paired-end reads. Fifth, it can check DNA strands. Sixth, it provides a function to convert alignment output to SAM format. Finally, its ACGT-count function is very convenient in that it produces the methylation ratios for all cytosines in a genome, thus it reduces the users’ time and effort needed to parse the large alignment output files.

Step 4: Examine bisulfite rates using nonCGc sites. For mammalian cells, the nonCGc sites (i.e., the cytosines that are not in a C_p_G pair) are highly likely to be un-methylated, so we choose to examine bisulfite conversion rates using these nonCGc sites. In this step, our pipeline can examine bisulfite rates at both the chromosome level and the target-region level (if target regions are provided). For the chromosome level analysis, our pipeline studies the distribution of all nonCGc sites using histograms and summary tables. For the target region analysis, instead of studying the bisulfite conversion of each individual nonCGc site, we summarize all the nonCGc sites within a target region. In particular, our pipeline calculates the mean and median bisulfite rates of all nonCGc sites with coverage for each region. It then generates summary tables and plots histograms for the statistical summary of all target regions.

If a dataset has very high bisulfite-conversion rates (i.e., > 0.999) as shown in the summary tables and plots, the user can continue to do further downstream analysis. However, if the results of this step show that the dataset has very low bisulfite conversion rates, the user may continue with caution. For example, if there is a large percentage (e.g., >30%) of nonCGc sites with very low bisulfite conversion rates, the users may have to further investigate their sequencing experiments to understand the problem of bisulfite conversion, or even discard the data. If there is a small proportion (e.g., <5%) of nonCGc sites with low bisulfite conversion rates, the user may split all nonCGc sites into two groups: (A) nonCGc sites with high bisulfite rates (e.g., >= 0.99) and (B) nonCGc sites with low bisulfite rates (e.g., < 0.99). The user may only use the C_p_G sites near the nonCGc sites in group (A) to do downstream analysis.

Step 5: Compare sequence structures of different regions. It is important to be aware that many factors can affect the quality of sequencing and genomic regions may respond differently to these factors. For example, some regions have low bisulfite conversion, while other regions do not; some regions have low coverage, while other regions have high coverage. It is unclear how these differences are related to DNA sequence structure (e.g., GC contents and repetitive regions). In order to interpret a sequencing experiment, it is necessary to know which regions have high or low coverage. In this step, our pipeline takes user-provided target regions as an input file. The target regions can be a list of genes with start and end positions, a list of chromosome regions, or a list of C_p_G islands in which the user is interested. The regions with high and low metrics (i.e., coverage and bisulfite conversion) are defined below:

1) High bisulfite conversion region: if the median bisulfite conversion rate of all nonCGc sites in a target region is greater than or equal to *B*, this region is selected as a high bisulfite conversion region.

2) Low bisulfite conversion region: if the median bisulfite conversion rate of all nonCGc sites in a target region is less than or equal to *b*, this region is selected as a low bisulfite conversion region.

3) High coverage region: For a given target, let *N* be the number of nonCGc sites and *n* be the number of nonCGc sites with coverage in a target region. If *n/N >= L*, it is selected as a high coverage region.

4) Low coverage region: For a given target, let *N* be the number of nonCGc sites and *n* be the number of nonCGc sites with coverage in a target region. If *n/N < l,* it is selected as a low coverage region.

As for the above high and low metric (i.e., coverage and bisulfite conversion) regions, we recommend the users first check the number of target regions in each group. If there are only a small number of regions (e.g., less than 10 target regions, or less than 0.5% of the total target regions) with low metric status, that means there may not be a serious coverage or bisulfite conversion issue. It is not necessary to compare the DNA sequence structure of high and low metric regions. The sample is probably very well sequenced. If, indeed, there are a large number of regions with low metric status, we recommend the users check further.

In order to investigate whether the coverage difference and bisulfite conversion problem are due to DNA sequence structures, our pipeline produces regions with low or high metrics as defined above, and then compares the DNA sequence structure of different regions. In particular, our pipeline generates plots for the percentage of A, C, G, T, C+G, CGc, nonCGc, and repetitive bases (i.e., “%low_count” provided by the UCSC genome browser) for these different regions.

Generally speaking, if the coverage differences (or bisulfite conversion problems) are not associated with DNA sequence structures, we will not see any dramatic differences when comparing the percentage of A, C, G, T, C+G, CGc, nonCGc, and repetitive bases for high and low coverage regions (or high and low bisulfite conversion regions). However, if we see some dramatic differences in the comparison plots, this may provide us some insight into the sequencing experiments. For example, if we see that the high coverage regions tend to have much lower percentages of GC contents (or nonCGc) and higher percentages of As or Ts, while low coverage regions tend to have the reverse patterns, this may indicate some bisulfite conversion problem. This problem is likely because bisulfite conversion may damage DNA fragments, leaving them broken and unable to be sequenced. In addition, if we find that the high and low coverage regions correspond to low and high “%low_count” (i.e., repetitive regions) respectively, this may indicate that the repetitive regions are not well sequenced. In the user manual (see the Additional file 1), we have provided different examples to illustrate our pipeline in more details.

The above are the five steps of the complete pipeline MethyQA. If users are familiar with alignment and have obtained the methylation ratios using either the BRAT ACGT-count program or some other alignment tools, they can skip Steps 1 to 3 and only use the partial pipeline provided in our package (named partial.MethyQA) to achieve the quality assessment in Steps 4 and 5. The BRAT methylation ratio output contains the following basic and standard information for each cytosine site: chromosome, position, cytosine type (i.e., CG, CHH, and CHG), total coverage, and methylation ratio. If users have used other alignment tools, as long as the output of these bisulfite sequencing alignment tools generate the above basic information, the output can be easily converted by switching the order of columns to the BRAT methylation ratio output format, then run our partial.MethyQA pipeline.

### Input and output

Our pipeline uses the raw FASTQ file as input in Step 1 and Step 2. In Steps 3, 4 and 5, the input files are the output files from the previous step. If the user is interested in studying specific target regions in Steps 4 and 5, a target file with three columns including chromosome, start and end positions for each region is required. As for the output, see Table 1 for a list of the main output files in each step of the MethyQA pipeline. In addition, the output files for Steps 1, 2 and 3 are well described in the FastQC and BRAT documentation files and details can be found there. More details about the input and output files are provided in the MethyQA user manual (see the Additional file 1).

**Table 1:** The main output files in each step of the MethyQA pipeline

**File name**	**Pipeline step**	**Descriptions**
SampleName_fastqc	Step 1	One folder and one zip file that save the output of quality assessment using fastqc.
SampleName_fastqc.zip		
fastx.trim.fastq	Step 2	Fastx or cutadapt output (one line per read) if adapter trimming is used.
cutadapt.trim.fastq		
*_reads1.txt	Step 2	BRAT trimming output (one line per read) if dynamic trimming using BRAT trim is done.
fixedTrim_BRATout	Step 2	Fixed length trimming output (one line per read) if “fixed-length” trimming is used.
alignment.brat	Step 3	BRAT alignment output (one line per read).
* _forw.txt	Step 3	BRAT ACGT-count (i.e., methylation ratio) output file (one line per cytosine position).
*chrN.summary.table.txt	Step 4	Chromosome level summary table for bisulfite conversion rates.
*chrN.BS.ps	Step 4	Chromosome level plot for bisulfite conversion rates.
*chrN.target.summary.table.txt	Step 4	Target region level summary table for the mean and median of bisulfite conversion rates.
*chrN.mean.median.ps	Step 4	Target region level plot for the mean and median of bisulfite conversion rates.
*chrN.seq.bisulfite.boxplot.ps	Step 5	Plots for comparing the DNA sequence structure for regions with high and low bisulfite conversion rates.
*chrN.highBS.seq	Step 5	Target regions with high or low bisulfite conversion rates (*seq files include all basic DNA sequence statistics, and *target files include the summary of nonCGc bisulfite conversion rates).
*chrN.lowBS.seq		
*chrN.highBS.target		
*chrN.lowBS.target		
*chrN.seq.coverage.boxplot.ps	Step 5	Plots for comparing the DNA sequence structure for regions with high and low sequencing coverage.
*chrN.highCoverage.seq	Step 5	Target regions with high or low sequencing coverage (*seq files include all basic DNA sequence statistics, and *target files include the summary of nonCGc bisulfite conversion rates).
*chrN.lowCoverage.seq		
*chrN.highCoverage.target		
*chrN.lowCoverage.target		

“*” means the prefix provided by the user while running MethyQA. In some file names, “chrN” means a specific chromosome that the user investigates.

### Usage and command options

Our pipeline is written in Perl and R. It can be run as shown below under a LINUX or UNIX environment.

The usage of the complete pipeline MethyQA is:

 perl MethyQA.pl -i <FASTQ_input> -t <TARGET_input> -c <chr> -p <prefix> -d <path_MethyQA> -R <reference_directory> -r <reference_name> [OPTIONS]

The command options of MethyQA are explained in Table 2.

**Table 2:** The command options of MethyQA

[-i <file>]	FASTQ input file
[-t <file>]	Target input file (i.e., a list of target regions specified for analysis). “F”, if do not perform target analysis
[-d <dir>]	Path to MethyQA directory (e.g., /home/user/downloads/MethyQA/)
[-c <string>]	Chromosome number (e.g., chr1, chr2, chr17, chrX, chrY, etc.)
[-p <string>]	Prefix (i.e., the prefix written to the output file names)
[-R <dir>]	Reference directory (i.e., the directory with the genome reference files)
[-r <file>]	Reference name (i.e., the file name of the reference that the user will use)
[-f <string>]	FASTQ format (i.e., “sanger” or “illumina”)
[-a <string>]	Adapter trimming. (1) “no”: no adapter trimming (default); (2) “fastx”: fastx adapter trimming; (3) “cutadapt”: cutadapt adapter trimming. If cutadapt is set, the “-Y” option should be specified in the command line
[-A <string>]	Adapter sequence (The default is Illumina adapter sequence: AGATCGGAAGAGCGGTTCAGCAGGAATGCCGAG)
[-T <string>]	Quality trim flag. (1) “ no”: no quality trimming; (2) “brat”: brat dynamic trimming (default); (3) “fix”: fixed quality trimming
[-N <int>]	For fixed quality trimming (users specify the number of bases to be trimmed at the 5' end, default is 5)
[-n <int>]	For fixed quality trimming (users specify the number of bases to be trimmed at the 3' end, default is 10)
[-B <real>]	Cutoff value for selecting high bisulfite conversion regions (Range: [0, 1], default B=0.99)
[-b <real>]	Cutoff value for selecting low bisulfite conversion regions (Range: [0, 1], default b=0.6)
[-L <real>]	Cutoff value for selecting high coverage region (Range: [0, 1], default L=0.5)
[-l <real>]	Cutoff value for selecting low coverage region (Range: [0, 1], default l=0.1)
[-u <logic>]	Bisulfite flag (it is an option to initiate boxplot of high vs. low bisulfite rates, either ‘TRUE’ (default) or ‘FALSE’)
[-v <logic>]	Coverage flag (it is an option to initiate boxplot of high vs. low coverage, either ‘TRUE’ (default) or ‘FALSE’)
[-Y <string>]	Path to python when running cutadapt (i.e., python, python2.6, /home/bin/python)
[-Q <string>]	Path to FastQC (e.g., /home/appl/apps/bin/fastqc, default is to use the one complied in MethyQA pipeline)
[-M <string>]	Path to BRAT trim function (e.g., /home/appl/apps/bin/trim.v1.2.4, default is to use the one complied in MethyQA pipeline)
[-K <string>]	Path to BRAT-large function (e.g., /home/appl/apps/bin/brat-large.v1.2.4, default is to use the one complied in MethyQA pipeline)
[-J <string>]	Path to BRAT ACGT-count function (e.g., /home/appl/apps/bin/acgt-count.v1.2.4, default is to use the one complied in MethyQA pipeline)
[-X <string>]	Path to fastx function (e.g., home/appl/apps/bin/fastx, default is to use the one complied in MethyQA pipeline)
[-C <string>]	Path to cutadapt function (e.g., /home/appl/apps/bin/cutadapt, default is to use the one complied in MethyQA pipeline)

The usage of the partial pipeline partial.MethyQA is:

 perl partial.MethyQA.pl -i <BASE_input> -t <TARGET_input> -c <chr> -p <prefix> -d <path_MethyQA> -R <reference_directory> [OPTIONS]

The command options of partial.MethyQA are similar to the complete pipeline MethyQA, and more details about these options are provided in the user manual (see the Additional file 1).

## Results

**Figure 2:**
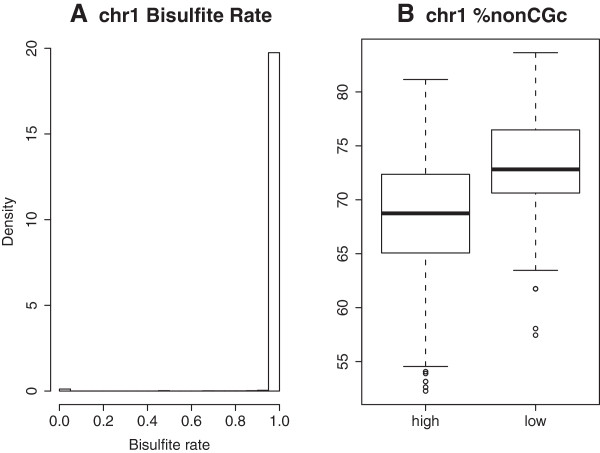
**Plots of bisulfite conversion rate (A) and nonGCc content (B).** Plot A is the histogram of the bisulfite conversion rates of nonCGc sites on chr1. Plot B is the non-CGc content in the high and low coverage regions.

We demonstrate the use of MethyQA using a publicly available bisulfite-treated methylation sequencing dataset for the cell line MCF10A [26]. Because the first three steps are conducted using available software packages, we mainly show the results of Steps 4 and 5. The reads in this dataset have low quality at the 3’ end. After trimming, about 1.5 million reads (2.5% of the total) that were thrown away from the raw data are aligned in the trimmed data (using the reference genome hg18). Thus, we use the alignment results obtained with low quality bases trimmed. Figure 2A is the bisulfite conversion (i.e., 1 - methylation ratio) rate of nonCGc sites in chr1. This figure shows that all data points are around 1, that is, the bisulfite conversion rate is very high and there is no evidence of incomplete conversion. In addition to the graphical summary, our pipeline also provides a summary table for chromosome level analysis (see Table 3). Table 3 shows that the total number of nonCGc sites on chr1 (TNCGC) is 44683043, and 622926 of them (i.e., 1.394%) have at least 1X coverage. The bisulfite conversion rates of more than 75% of the nonCGc sites are 100%. In combination with the Figure 2A, the examination results show that this dataset has very high bisulfite conversion rate. If a dataset has low bisulfite conversion rates, the histogram will be very different from the above one, that is, there will be data points with values much less than 1. In the user manual (see the Additional file 1), we provide different examples of datasets with and without problems.

**Table 3:** Example of bisulfite-rate summary at the chromosome level

**chr**	**TNCGC**	**TNCGCwC**	**Percent**	**Min**	**25**^**th**^**pecentile**	**Median**	**Mean**	**75**^**th**^**perecentile**	**Max**
Chr1	44683043	622926	1.394%	0	1	1	0.9961	1	1

TNCGC means the “total number of nonCGc sites”. TNCGCwc means the “total number of nonCGc sites with coverage”. “Percent” means the percent of nonCGc sites with coverage. The last 6 columns are a 6-number-summary (minimum, 25th percentile, median, mean, 75th percentile, and maximum) for the bisulfite rates of nonCGc sites on chr1.

**Figure 3:**
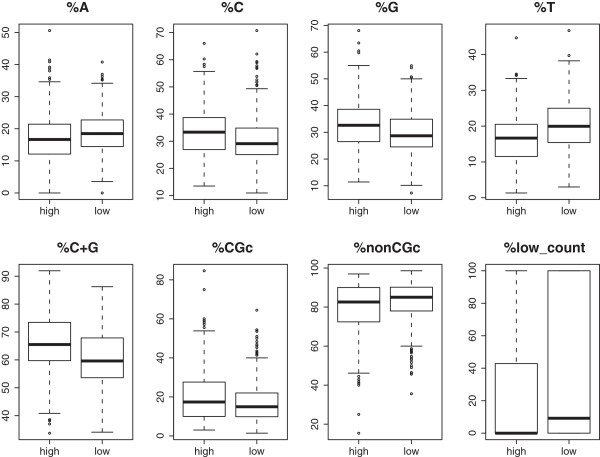
**Plots of comparing regions with high and low coverage.** The comparison is based on the percentages of A, C, G, T, GC content (i.e., C+G), CGc, nonCGc, and repetitive bases.

Figure 2B compares the percentage of nonCGc sites for regions with high and low coverage. This figure shows that low coverage regions tend to have higher nonGCc content than high coverage regions. In addition to comparing the nonCGc proportions, our pipeline can compare the DNA sequence structures of high or low coverage (or bisulfite conversion rate) regions in more detail as explained in Step 5 of our pipeline. For example, we may compare the DNA sequence structure for high coverage with low coverage target regions (see Figure 3). In Figure 3, we use the genomic regions obtained based on the RRBS protocol as target regions because this MCF10A sample is sequenced using the RRBS method. In particular, we use the chromosome regions (or intervals) obtained with the MspI (C^CGG) sites and within 40~220 base-distance. Figure 3 compares the percentages of A, C, G, T, GC content (i.e., C+G), CGc, nonCGc, and repetitive bases in high-coverage regions with the ones in low coverage regions. From this figure, we see that there is no obvious difference between high and low coverage region, which is because this sample is well sequenced and there is no obvious sequencing problem. However, for some datasets that may have known or unknown library preparation or sequencing problems, the DNA sequence structure plots generated in Step 5 will show obvious patterns. For example, some data will show high coverage corresponding to dramatically high or low percentages of A, GC, or nonCGc contents, and so on. More information about other examples and our pipeline can be found in the user manual (see the Additional file 1).

## Discussion

Our pipeline has a few limitations. First, for some non-mammalian genomes (e.g., plants), nonCGc sites are methylated. Our pipeline will not be suitable for checking the bisulfite–treated methylation sequencing data generated from these genomes. For these genomes, the investigator may use some positive and negative controls (e.g., some regions or sites known to be methylated or unmethylated). Then the users may study the methylation ratios of these known regions. Second, our pipeline is mainly developed for the FASTQ format sequencing data generated using the Illumina analyzer. Sequencing data that are not in the FASTQ file format first need to be converted to a FASTQ file in order to use our MethyQA program. Despite these limitations, the perl and R scripts provided by our group can be used to conduct further analysis with pre-obtained methylation ratios.

Our pipeline has the following advantages. First, because our pipeline is not designed for specific protocol generated data, it is suitable for performing quality assessment for bisulfite sequencing data generated by different protocols. Second, the user can conduct the quality assessment, not only at the individual chromosome level, but also at a user-provided target-region level. If the users are interested in whole genome sequencing or checking bisulfite conversion rates, they can utilize the chromosome level analysis. In contrast, if users are mainly interested in certain type of regions (e.g., CpG islands, promoter regions, or candidate genes), the target-region-analysis feature can be utilized as it allows the users to focus on specific regions of interest.

## Conclusions

The development of pipelines for bisulfite-treated methylation sequencing quality data is highly needed. MethyQA is a new tool that can fill this need. It can process large amounts of raw and aligned methylation sequencing data efficiently. It generates both diagnostic graphs and tables to examine sequencing quality, providing useful information for medical researchers and analysts.

## Availability and requirements

**Project name:** MethyQA

**Downloading software (pipeline):**http://hal.case.edu/~sun/MethyQA.v2.zip

**Operating system(s):** Linux/Unix

**Programming language:** Perl (v5.8 or later), R (v2.13 or later) and Python (v2.6 or later)

**Other requirements:** None

**Any restrictions to use by non-academics:** None

## Competing interests

The authors declare that they have no competing interests.

## Authors' contributions

SS and AN wrote the perl and R scripts. XY provided original alignment scripts and helped with revising the final scripts. All three authors have been involved in the writing of manuscript and approved the final document.

## Supplementary Material

Additional file 1 MethyQA User Manual. Click here for file

## References

[B1] Chung W Kwabi-Addo B Ittmann M Jelinek J Shen L Yu Y Issa JP Identification of novel tumor markers in prostate, colon and breast cancer by unbiased methylation profiling PLoS One 2008 3 4 e2079 10.1371/journal.pone.0002079 18446232 PMC2323612

[B2] Fiegl H Millinger S Goebel G Muller-Holzner E Marth C Laird PW Widschwendter M Breast cancer DNA methylation profiles in cancer cells and tumor stroma: association with HER-2/neu status in primary breast cancer Cancer Res 2006 66 1 29 33 10.1158/0008-5472.CAN-05-2508 16397211

[B3] Huang TH Perry MR Laux DE Methylation profiling of CpG islands in human breast cancer cells Hum Mol Genet 1999 8 3 459 470 10.1093/hmg/8.3.459 9949205

[B4] Khalili A Potter D Yan P Li L Gray J Huang T Lin S Gamma-normal-gamma mixture model for detecting differentially methylated Loci in three breast cancer cell lines Cancer Inform 2007 3 43 54 19455234 PMC2675845

[B5] Lin HJ Zuo T Lin CH Kuo CT Liyanarachchi S Sun S Shen R Deatherage DE Potter D Asamoto L Breast cancer-associated fibroblasts confer AKT1-mediated epigenetic silencing of Cystatin M in epithelial cells Cancer Res 2008 68 24 10257 10266 10.1158/0008-5472.CAN-08-0288 19074894 PMC2821873

[B6] Tan LW Bianco T Dobrovic A Variable promoter region CpG island methylation of the putative tumor suppressor gene Connexin 26 in breast cancer Carcinogenesis 2002 23 2 231 236 10.1093/carcin/23.2.231 11872627

[B7] Widschwendter M Jones PA DNA methylation and breast carcinogenesis Oncogene 2002 21 35 5462 5482 10.1038/sj.onc.1205606 12154408

[B8] Yan PS Perry MR Laux DE Asare AL Caldwell CW Huang TH CpG island arrays: an application toward deciphering epigenetic signatures of breast cancer Clin Cancer Res 2000 6 4 1432 1438 10778974

[B9] Yang X Yan L Davidson NE DNA methylation in breast cancer Endocr Relat Cancer 2001 8 2 115 127 10.1677/erc.0.0080115 11446343

[B10] Zhu W Qin W Hewett JE Sauter ER Quantitative evaluation of DNA hypermethylation in malignant and benign breast tissue and fluids Int J Cancer 2010 126 2 474 482 10.1002/ijc.24728 19618401 PMC3398695

[B11] Kim MS Lee J Sidransky D DNA methylation markers in colorectal cancer Cancer Metastasis Rev 2010 29 1 181 206 10.1007/s10555-010-9207-6 20135198

[B12] Lin J Lai M Huang Q Ma Y Cui J Ruan W Methylation patterns of IGFBP7 in colon cancer cell lines are associated with levels of gene expression J Pathol 2007 212 1 83 90 10.1002/path.2144 17334979

[B13] Toyota M Ahuja N Ohe-Toyota M Herman JG Baylin SB Issa JP CpG island methylator phenotype in colorectal cancer Proc Natl Acad Sci U S A 1999 96 15 8681 8686 10.1073/pnas.96.15.8681 10411935 PMC17576

[B14] Zitt M Muller HM DNA methylation in colorectal cancer–impact on screening and therapy monitoring modalities? Dis Markers 2007 23 1–2 51 71 17325426 10.1155/2007/891967 PMC3851076

[B15] Sun S Chen Z Yan PS Huang YW Huang TH Lin S Identifying hypermethylated CpG islands using a quantile regression model BMC Bioinforma 2011 12 54 10.1186/1471-2105-12-54 PMC3051900 21324121

[B16] Esteller M Corn PG Baylin SB Herman JG A gene hypermethylation profile of human cancer Cancer Res 2001 61 8 3225 3229 11309270

[B17] Suijkerbuijk KP van Diest PJ van der Wall E Improving early breast cancer detection: focus on methylation Ann Oncol 2011 22 1 24 29 10.1093/annonc/mdq305 20591821

[B18] Strathdee G Brown R Aberrant DNA methylation in cancer: potential clinical interventions Expert Rev Mol Med 2002 4 4 1 17 14987388 10.1017/S1462399402004222

[B19] Wei SH Brown R Huang TH Aberrant DNA methylation in ovarian cancer: is there an epigenetic predisposition to drug response? Ann N Y Acad Sci 2003 983 243 250 10.1111/j.1749-6632.2003.tb05979.x 12724229

[B20] Widschwendter M Menon U Circulating methylated DNA: a new generation of tumor markers Clin Cancer Res 2006 12 24 7205 7208 10.1158/1078-0432.CCR-06-2531 17189390

[B21] Cokus SJ Feng S Zhang X Chen Z Merriman B Haudenschild CD Pradhan S Nelson SF Pellegrini M Jacobsen SE Shotgun bisulphite sequencing of the Arabidopsis genome reveals DNA methylation patterning Nature 2008 452 7184 215 219 10.1038/nature06745 18278030 PMC2377394

[B22] Lister R O'Malley RC Tonti-Filippini J Gregory BD Berry CC Millar AH Ecker JR Highly integrated single-base resolution maps of the epigenome in Arabidopsis Cell 2008 133 3 523 536 10.1016/j.cell.2008.03.029 18423832 PMC2723732

[B23] Meissner A Mikkelsen TS Gu H Wernig M Hanna J Sivachenko A Zhang X Bernstein BE Nusbaum C Jaffe DB Genome-scale DNA methylation maps of pluripotent and differentiated cells Nature 2008 454 7205 766 770 18600261 10.1038/nature07107 PMC2896277

[B24] Lister R Pelizzola M Kida YS Hawkins RD Nery JR Hon G Antosiewicz-Bourget J O'Malley R Castanon R Klugman S Hotspots of aberrant epigenomic reprogramming in human induced pluripotent stem cells Nature 2011 471 7336 68 73 10.1038/nature09798 21289626 PMC3100360

[B25] Lister R Pelizzola M Dowen RH Hawkins RD Hon G Tonti-Filippini J Nery JR Lee L Ye Z Ngo QM Human DNA methylomes at base resolution show widespread epigenomic differences Nature 2009 462 7271 315 322 10.1038/nature08514 19829295 PMC2857523

[B26] Sun Z Asmann YW Kalari KR Bot B Eckel-Passow JE Baker TR Carr JM Khrebtukova I Luo S Zhang L Integrated analysis of gene expression, CpG island methylation, and gene copy number in breast cancer cells by deep sequencing PLoS One 2011 6 2 e17490 10.1371/journal.pone.0017490 21364760 PMC3045451

[B27] Hansen KD Timp W Bravo HC Sabunciyan S Langmead B McDonald OG Wen B Wu H Liu Y Diep D Increased methylation variation in epigenetic domains across cancer types Nat Genet 2011 43 8 768 775 10.1038/ng.865 21706001 PMC3145050

[B28] Brunner AL Johnson DS Kim SW Valouev A Reddy TE Neff NF Anton E Medina C Nguyen L Chiao E Distinct DNA methylation patterns characterize differentiated human embryonic stem cells and developing human fetal liver Genome Res 2009 19 6 1044 1056 10.1101/gr.088773.108 19273619 PMC2694474

[B29] Chen PY Cokus SJ Pellegrini M BS Seeker: precise mapping for bisulfite sequencing BMC Bioinforma 2010 11 203 10.1186/1471-2105-11-203 PMC2871274 20416082

[B30] Harris EY Ponts N Levchuk A Roch KL Lonardi S BRAT: bisulfite-treated reads analysis tool Bioinformatics 2010 26 4 572 573 10.1093/bioinformatics/btp706 20031974 PMC3716225

[B31] Xi Y Li W BSMAP: whole genome bisulfite sequence MAPping program BMC Bioinforma 2009 10 232 10.1186/1471-2105-10-232 PMC2724425 19635165

[B32] Pedersen B Hsieh TF Ibarra C Fischer RL MethylCoder: software pipeline for bisulfite-treated sequences Bioinformatics 2011 27 17 2435 2436 10.1093/bioinformatics/btr394 21724594 PMC3157921

[B33] Sun Z Baheti S Middha S Kanwar R Zhang Y Li X Beutler AS Klee E Asmann YW Thompson EA SAAP-RRBS: Streamlined Analysis and Annotation Pipeline for Reduced Representation Bisulfite Sequencing Bioinformatics 2012 28 16 2180 2181 10.1093/bioinformatics/bts337 22689387 PMC3413387

[B34] Gu H Smith ZD Bock C Boyle P Gnirke A Meissner A Preparation of reduced representation bisulfite sequencing libraries for genome-scale DNA methylation profiling Nat Protoc 2011 6 4 468 481 10.1038/nprot.2010.190 21412275

[B35] Ecker JR Zeroing in on DNA methylomes with no BS Nat Methods 2010 7 435 437 10.1038/nmeth0610-435 20508637

[B36] Andrews S FastQC 2010 http://www.bioinformatics.bbsrc.ac.uk/projects/fastqc/

[B37] Yu X Guda K Willis J Veigl M Wang Z Markowitz S Adams M Sun S How well do alignment programs perform on sequencing data with varying qualities and from repetitive regions? BioData Min 2012 5 6 10.1186/1756-0381-5-6 22709551 PMC3414812

[B38] Hannon GJ FASTX-Toolkit 2009 http://hannonlab.cshl.edu/fastx_toolkit/

[B39] Martin M Cutadapt removes adapter sequences from high-throughput sequencing reads EMBnetjournal 2011 17 1 10 12

[B40] Krueger F Andrews SR Bismark: a flexible aligner and methylation caller for Bisulfite-Seq applications Bioinformatics 2011 27 11 1571 1572 10.1093/bioinformatics/btr167 21493656 PMC3102221

[B41] Langmead B Trapnell C Pop M Salzberg SL Ultrafast and memory-efficient alignment of short DNA sequences to the human genome Genome Biol 2009 10 3 R25 10.1186/gb-2009-10-3-r25 19261174 PMC2690996

[B42] Wu TD Nacu S Fast and SNP-tolerant detection of complex variants and splicing in short reads Bioinformatics 2010 26 7 873 881 10.1093/bioinformatics/btq057 20147302 PMC2844994

[B43] Harris EY Ponts N Le Roch KG Lonardi S BRAT-BW: efficient and accurate mapping of bisulfite-treated reads Bioinformatics 2012 28 13 1795 1796 10.1093/bioinformatics/bts264 22563065 PMC3381974

