# Supplementary material for: MethyQA: a pipeline for bisulfite-treated methylation sequencing quality assessment
Source: BMC Bioinformatics. 2013 Aug 23;14:259. doi: 10.1186/1471-2105-14-259 (PMC3765750; doi:10.1186/1471-2105-14-259)
Supplement: Additional file 1 — MethyQA User Manual. [file 1471-2105-14-259-S1.pdf]

MethyQA User Manual

Shuying Sun (ssun5211@yahoo.com)

Aaron Noviski (ajn21@case.edu)

Xiaoqing Yu (xxy39@case.edu)

June 5, 2013

Case Western Reserve University

Contents

1. General Overview and Installation — Page 2

1.1 General Overview — Page 2

1.2 Installation — Page 3

2. Pipeline Walkthrough — Page 4

2.1 Usage — Page 4

2.2 Initial Input — Page 7

2.3 Step 1 – FastQC — Page 9

2.4 Step 2 – Trimming Sequence Data — Page 9

2.5 Step 3 – BRAT alignment and ACGT-count — Page 10

2.6 Step 4.1 – Data Parser — Page 11

2.7 Step 4.2 – Chromosome-level Analysis — Page 11

2.8 Step 4.3 – Target-region Analysis — Page 13

2.9 Step 5 – Sequence Structure Analysis — Page 15

3. Additional Notes — Page 19

3.1 System Requirements — Page 19

3.2 Running Time — Page 19

3.3 Example — Page 19

3.4 Warning and troubling shooting notes — Page 19

4. References — Page 20

1. General Overview and Installation

1.1 General Overview

The MethyQA pipeline is meant to assist the user in assessing the quality of bisulfite-treated methylation sequencing data. It achieves this goal by utilizing efficient perl and R scripts to first parse the data and then summarize them in useful formats. To execute the process, the user enters a command line in a Unix/Linux environment and the pipeline will begin and update the user with the overall progress of the assignment. By the end of the process, the user receives summary tables and plots analyzing bisulfite conversion rates at the chromosome-level as well as at the target-region level (the target input file is to be provided by the user). The user will also obtain graphical summaries analyzing the sequence structure for regions with high vs. low coverage and bisulfite conversion rates. This pipeline is developed with the aim of providing insights for experimental scientists in a timely manner. In particular, our pipeline can provide quality assessment for tens of millions of reads in less than one hour. The workflow of the pipeline is illustrated below.

Workflow of the Pipeline MethyQA

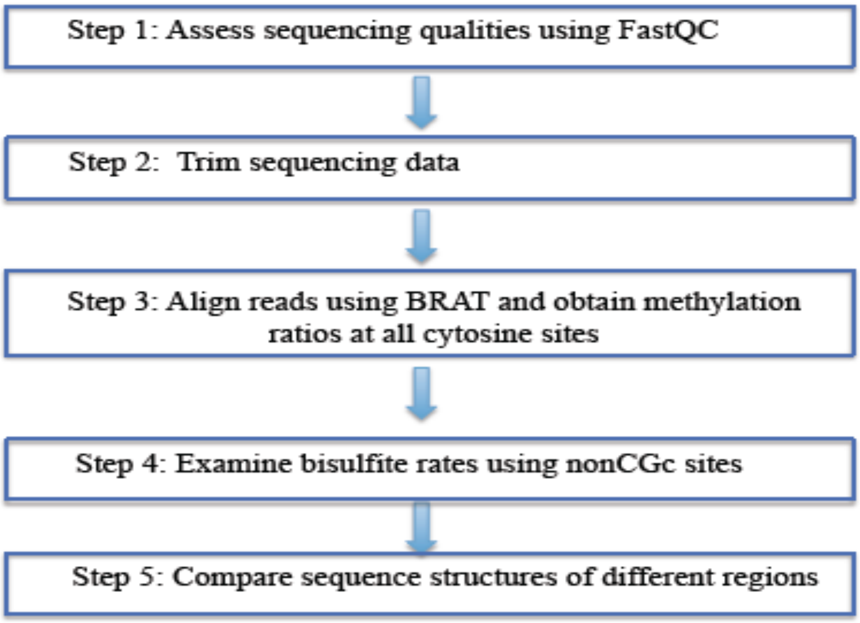

Note, the last two steps were developed by our research team utilizing various scripts included in the MethyQA code file.

We demonstrate the use of MethyQA using a publicly available bisulfite-treated methylation sequencing dataset (GSE27003) for the cell line MCF10A and another dataset named “s7” in section 2. MCF10A data is considered to be the example of a “good” dataset with no obvious sequencing problems and “s7” is considered to be a ”bad” dataset that has a low bisulfite conversion rate.

1.2 Installation

Perl (recommend v5.8.8 or later), R (recommend v2.13.0 or later), and python (recommended v2.6 or later) are required on the user's system. Then the user can simply download the MethyQA package, and after unzipping, it is ready to go, because FastQC, BRAT, Fastx, and Cutadapt are already pre-compiled in the MethyQA resources folder.

1.2.1 Installing MethyQA

MethyQA requires a Linux/Unix system, with proper versions of Perl, R, and python installed. To install MethyQA, the user can download the pipeline from: http://hal.case.edu/~sun/MethyQA.v2.zip. After unzipping the file ("unzip MethyQA.v2.zip"), the user gets a folder include 2 documents and 3 subfolders:

MethyQA.user.manual.pdf: a copy of this user manual.
README.txt: about how to unzip, install, and run MethyQA pipeline (with detailed example scripts)
Code: a folder containing all perl and R scripts used for MethyQA pipeline.
Input: a folder containing all example input data as mentioned in README.txt.
Resource: a folder containing all third-party software packages that are already pre-compiled in the MethyQA, including FastQC, BRAT, Fastx, and Cutadapt.

Now, it is ready to go. If the user wishes to download the third-party software separately, he can follow the tutorial provided below (1.2.2 – 1.2.5).

1.2.2 Installing FastQC

To install FastQC, the user should go to: http://www.bioinformatics.babraham.ac.uk/projects/fastqc/. Then the user needs to unzip the package and the latest version of FastQC will be ready to run.

1.2.3 Installing BRAT

Installing BRAT is a fairly simple process. After unzipping the BRAT package, the user only needs to run 'make' to build executable files. An example follows:

```
$ wget http://compbio.cs.ucr.edu/brat/downloads/brat-1.2.3.tar.gz
$ tar zxvf brat-1.2.3.tar.gz
$ cd brat-1.2.3
$ make
```

For additional information the user can learn more on BRAT's website: http://compbio.cs.ucr.edu/brat/

1.2.4 Installing Cutadapt

The user can choose to utilize Cutadapt's adapter trimming function, or Fastx-clipper's adapter trimmer (or neither of them). If the user chooses to go with Cutadapt, it should be a fairly simple installation. The user must have Python 2.6, and then easy_install can be used to quickly install Cutadapt. The only command needed follows:

$ easy_install cutadapt

Additional help can be found at: http://code.google.com/p/cutadapt/

1.2.5 Installing Fastx

Installing Fastx is a bit more involved than BRAT and Cutadapt because it involves additional software to work. For more details the user can visit: http://hannonlab.cshl.edu/fastx_toolkit/index.html

To start, the user needs wget, pkg-config, g/g++ compiler, and libgtestutils-0.6. The script to install libgtextutils is below:

```
$ wget http://cancan.cshl.edu/labmembers/gordon/files/libgtextutils-0.6.tar.bz2
$ tar -xjf libgtextutils-0.6.tar.bz2
$ cd libgtextutils-0.6
$ ./configure
$ make
$ sudo make install // this part is optional

//Tell pkg-config to look for libraries in /usr/local/lib, too.
$ export PKG_CONFIG_PATH=/usr/local/lib/pkgconfig:$PKG_CONFIG_PATH
```

Now to install fastx-toolkit, the user may copy the following command lines:

```
$ wget http://cancan.cshl.edu/labmembers/gordon/files/fastx_toolkit-0.0.12.tar.bz2
$ tar -xjf fastx_toolkit-0.0.12.tar.bz2
$ cd fastx_toolkit-0.0.12
$ ./configure
$ make
$ sudo make install
```

After following the above steps, the software packages will work. If the user needs any more help, please refer to the respective websites listed above.

2. Pipeline Walkthrough

2.1 Usage

2.1.1 Using the complete pipeline

The complete pipeline runs analysis for the workflow step 1-5 as shown in 1.1 with the following usage.

perl MethyQA.pl -i <FASTQ_input> -t <TARGET_input> -c <chr> -p <prefix> -d <path_MethyQA> -R <reference_directory> -r <reference_name> [OPTIONS]

Command options:

[-i <file>]	FASTQ input file
[-t <file>]	Target input file (i.e., a list of target regions specified for analysis). "F", if the users do not perform target analysis
[-d <dir>]	Path to MethyQA directory (e.g., /home/user/downloads/MethyQA/)
[-c <string>]	Chromosome number (e.g., chr1, chr2, chr17, chrX, chrY, etc.)
[-p <string>]	Prefix (i.e., the prefix written to the output file names)
[-R <dir>]	Reference directory (i.e., the directory with the genome reference files)
[-r <file>]	Reference name (i.e., the file name of the reference that the user will use)
[-f <string>]	FASTQ format (i.e., "sanger" or "illumina")
[-a <string>]	Adapter trimming. (1) "no": no adapter trimming (default); (2) "fastx": fastx adapter trimming; (3) "cutadapt": cutadapt adapter trimming. If cutadapt is set, the "-Y" option needs to be specified in the command line
[-A <string>]	Adapter sequence (the default is Illumina adapter sequence: AGATCGGAAGAGCGGTTCAGCAGGAATGCCGAG)
[-T <string>]	Quality trim flag. (1) " no": no quality trimming; (2) "brat": brat dynamic trimming (default); (3) "fix": fixed quality trimming
[-N <int>]	For fixed quality trimming (the users specify the number of bases to be trimmed at the 5' end, default is 5)
[-n <int>]	For fixed quality trimming (the users specify the number of bases to be trimmed at the 3' end, default is 10)
[-B <real>]	Cutoff value for selecting high bisulfite conversion regions (Range: [0, 1], default B=0.99) (Note: if the median bisulfite conversion rate of all nonCGc sites in a target region is greater than or equal to B, this region is elected as a high bisulfite conversion region)
[-b <real>]	Cutoff value for selecting low bisulfite conversion regions (Range: [0, 1], default b=0.6) (Note: if the median bisulfite conversion rate of all nonCGc sites in a target region is less than b, this region is elected as a low bisulfite conversion region)
[-L <real>]	Cutoff value for selecting high coverage region (Range: [0, 1], default L=0.5) (Note: Let N be the number of nonCGc sites and n be the number of nonCGc sites with coverage in a target region. For a given target, if n/N >= L, it is selected as a high coverage region)
[-l <real>]	Cutoff value for selecting low coverage region (Range: [0, 1], default l=0.1) (Note: Let N be the number of nonCGc sites and n be the number of nonCGc sites with coverage in a target region. For a given target, if n/N < l, it is selected as a low coverage region)
[-u <logic>]	Bisulfite flag (it is an option to initiate boxplot of high vs. low bisulfite rates, either 'TRUE' (default) or 'FALSE')
[-v <logic>]	Coverage flag (it is an option to initiate boxplot of high vs. low coverage, either 'TRUE' (default) or 'FALSE')
[-Y <string>]	Path to python when running cutadapt (i.e., python, python2.6, /home/bin/python)
[-Q <string>]	Path to FastQC (e.g., /home/appl/apps/bin/fastqc, default is to use the one complied in MethyQA pipeline)
[-M <string>]	Path to BRAT trim function (e.g., /home/appl/apps/bin/trim.v1.2.4, default is to use the one complied in MethyQA pipeline)
[-K <string>]	Path to BRAT-large function (e.g., /home/appl/apps/bin/brat-large.v1.2.4, default is to use the one complied in MethyQA pipeline)
[-J <string>]	Path to BRAT ACGT-count function (e.g., /home/appl/apps/bin/acgt-count.v1.2.4, default is to use the one complied in MethyQA pipeline)
[-X <string>]	Path to fastx function (e.g., home/appl/apps/bin/fastx, default is to use the one

complied in MethyQA pipeline)

[-C <string>] Path to cutadapt function (e.g., /home/appl/apps/bin/cutadapt, default is to use the one complied in MethyQA pipeline)

The complete pipeline ties the software and source code together with appropriate dataflow to ensure correct output is achieved. At this point, the user needs to have perl, R, FastQC, and BRAT software installed on their system and be able to enter commands in a Unix/Linux environment. The initial target and fastq input files will be discussed in section 2.2. The output directory is the path, specified by the user, to which the output files will be written. All output files will be written to the current working directory set by the user. The "-c" argument is used to specify one chromosome for analysis (i.e. "chr1", or "chr17", or "chrX", etc.). It is also important to note that for all chromosome positions, 1-based values are used throughout the pipeline.

Example command line (adapter trimming with cutadapt, target-region analysis on chr2)

perl /home/MethyQA/code/MethyQA.pl -i /home/ MethyQA/ input/sample.fastq -t /home/MethyQA/input/targets.file -d /home/MethyQA/ -c chr2 -p sample.name -R /home/reference/hg18/ -r /home/MethyQA/input/hg18.chr2.fa.filename.txt -a cutadapt -Y python2.6

2.1.2 Using the partial pipeline

If the users have already generated the BRAT alignment output file or have obtained the methylation ratio data using other alignment tools, they can start to run the analysis of Step 4 and 5 using our "partial.MethyQA" pipeline whose usage is given below.

perl partial.MethyQA.pl -i <BASE_input> -t <TARGET_input> -c <chr> -p <prefix> -d <path_MethyQA> -R <reference_directory> [OPTIONS]

Command options:

[-i <file>] Base input file (the BRAT ACGT-count output file)

[-t <file>] Target input file (i.e., a list of target regions specified for analysis). "F", if do not perform target analysis

[-d <dir>] Path to MethyQA directory (e.g., /home/user/downloads/MethyQA/)

[-c <string>] Chromosome number (e.g., chr1, chr2, chr17, chrX, chrY, etc.)

[-p <string>] Prefix (i.e., the prefix written to the output file names)

[-R <dir>] Reference directory (i.e., the directory with the genome reference files)

[-B <real>] Cutoff value for selecting high bisulfite conversion regions (Range: [0, 1], default B=0.99) (Note: if the median bisulfite conversion rate of all nonCGc sites in a target region is greater than or equal to B, this region is elected as a high bisulfite conversion region)

[-b <real>] Cutoff value for selecting low bisulfite conversion regions (Range: [0, 1], default b=0.6) (Note: if the median bisulfite conversion rate of all nonCGc sites in a target region is less than b, this region is elected as a low bisulfite conversion region)

[-L <real>] Cutoff value for selecting high coverage region (Range: [0, 1], default L=0.5) (Note: Let N be the number of nonCGc sites and n be the number of nonCGc sites with coverage in a target region. For a given target, if n/N >= L, it is selected as a high coverage region)

[-l <real>]	Cutoff value for selecting low coverage region (Range: [0, 1], default l=0.1) (Note: Let N be the number of nonCGc sites and n be the number of nonCGc sites with coverage in a target region. For a given target, if n/N < l, it is selected as a low coverage region)
[-u <logic>]	Bisulfite flag (it is an option to initiate boxplot of high vs. low bisulfite rates, either 'TRUE' (default) or 'FALSE')
[-v <logic>]	Coverage flag (it is an option to initiate boxplot of high vs. low coverage, either 'TRUE' (default) or 'FALSE')

As outlined in the manuscript, Steps 1-3 of the pipeline utilize available software packages to prepare the data for Steps 4 and 5. If a BRAT ACGT-count output file already exists, the user can opt to start the pipeline from Step 4, which is the start of the process developed by our research team. The ACGT-count input file will be discussed in section 2.2.

The users may have obtained the methylation ratios using some other alignment tools. In this case, they can still use partial.MethyQA. They just need to make sure the methylation ratio files are prepared to have the same format as BRAT output. The BRAT methylation ratio output contains the following basic and standard information for each cytosine site: chromosome, position, cytosine type (i.e., CG, CHH, and CHG), total coverage, and methylation ratio. For more details regarding BRAT methylation ratio output, see section 2.2.2.

Example command line (target-region analysis on chr2)

```
perl /home/MethyQA/code/partial.MethyQA.pl -i sample.acgt_forw.txt -t
/home/MethyQA/input/targets.file -d /home/MethyQA/ -c chr2 -p sample.name -R
/home/reference/hg18
```

2.2 Initial Input

2.2.1 FASTQ Input File

The FASTQ input file contains the raw data that will be analyzed in the upcoming steps. A sample of a FASTQ formatted file is shown below:

Box 1. FASTQ input file 1 read:

```
@methy.MCF10A.SRR097806.1 HWW-EAS217_0007:6:2:0:815 length=50
NGGTGTTTTTTGGGTTTTAGTAGTTNNGGTTCGTGGTTAGTNNGATTTGT
+methy.MCF10A.SRR097806.1 HWW-EAS217_0007:6:2:0:815 length=50
!.9768:;;;8547:;;;989526#!!##############!!#######
```

With FASTQ format, there should be 4 lines attributed for every read. The FASTQ input file is the direct input for Steps 1 and 2, and from there, the output files produced will be used as inputs for the remaining steps.

2.2.2 ACGT-count Input File (only used as initial input for the partial pipeline, see section 2.1.2)

The ACGT-count file is produced from the alignment software, BRAT. The sample file, shown in box 1, lists its position values in 0-based offset. Upon execution of Step 4 of the pipeline, the first process is to

separate the CG-type and Coverage columns by removing the ":" and changing the position values to 1-based offset.

Box 2

chr	position	position	CG-type:Coverage	Methylation	Strand
chr5	63004	63004	CHH:0	0	+
chr5	63005	63005	CHH:0	0	+
chr5	63006	63006	CHH:0	0	+

chr –chromosome number
position – 0-based chromosome position of a cytosine site
CG-type – the CG-type of a cytosine site, whether it is CG site or a nonCG site
coverage – number of reads
methylation – this column displays the methylation ratio, and the bisulfite rate at nonCGc sites can be obtained as bisulfite rate = 1-methylation ratio)
strand – chromosome strand ("+" or "-")

Note that the header of the sample was added in this document for clarification. The ACGT-count input has been well sorted in the original output as done by the BRAT software.

2.2.3 Target Input File

The target input is a list of target regions desired for analysis by the user. It includes the chr name, the start and end positions. While the position input file is produced from the BRAT software, the target input file is to be produced by the user. This is due to the fact that the user will provide specific target-regions to analyze. An example of a target file is shown in box 3. In this box, "chr" means the chromosome number; "start" means the start position of the target region to analyze; and "end" means the end position of the target region to analyze.

Box 3

chr	start	end
chr5	103805	104899
chr5	106111	107264

There could be additional columns in the target input file, however, the important parts are the first 3 columns, i.e. the chr, start, and end values. The target regions will be sorted numerically by chromosome and target-region start position in step 4.1 of the pipeline.

2.2.4 Chromosome List File

The chromosome list file shows the location of the reference genome for the chromosomes the users want to analyze.

Box 4

```
/home/reference/ hg18/chr5.fa
/home/reference/hg18/chr10.fa
```

2.3 Step 1: FastQC

FastQC is already detailed online, so this section will only briefly describe the input and output for this step of the pipeline. For more information, the documentation for the FastQC software is located at: www.bioinformatics.bbsrc.ac.uk/projects/fastqc/

2.3.1 FastQC input

The input for the FastQC software is a fastq file. This will be the initial input for the complete pipeline.

2.3.2 FastQC output

The output for the FastQC software will be packaged in a zip file and a folder in the output directory.

2.4 Step2: Trim

2.4.1 Adapter Trimming

An adapter trimming algorithm removes adapter sequences from high-throughput sequencing data. The user will be able to utilize either the adapter trimming tool *cutadapt* or the *fastx_clipper* by specifying which tool to use in the global variables section at the beginning of MethyQA.pl. The user could also choose to skip adapter trimming by entering a string "no", note that the default setting is no adapter trimming. For more information on cutadapt, the user can visit the website: code.google.com/p/cutadapt/. For more information on fastx_clipper, the user can visit the website: hannonlab.cshl.edu/fastx_toolkit/index.html

2.4.2 BRAT Trim

Documentation on the BRAT trim function can be found in the BRAT user manual, which can be found at the website: http://compbio.cs.ucr.edu/brat/.

2.4.2.1 BRAT Trim input

The input file for BRAT Trim is the initial FASTQ file used in step 1 of the pipeline. Although detailed information can be found in the BRAT user manual, we will briefly describe some of the default parameter settings in the pipeline as demonstrated in the following script line.

trim -s $ARGV[0] -P $pref -q 1 -L 64 -m 2;

In this command, "q" represents the quality score threshold of bases, "L" specifies the smallest value of the range of base quality scores in ASCII representation (64 for Illumina format fastq file and 33 for sanger format fastq file), and "m" is the number of allowable internal N's.

2.4.2.2 BRAT Trim output

There are a few parameter options that are discussed in the BRAT user manual (and the specific parameters we've implemented are briefly described in the source code notes). However, the output file important for the progression of the pipeline will be labeled "prefix_reads1.txt" and a sample is shown below in box 5.

Box 5

```
prefix_reads1.txt
GGGTTTGGTGGTTGGTATTTGTATGTAATTTTAGTTATTTGGGAGGTTG          1     0
ATCGGAAGAGCGGTTCAGCAGGAATGCCGAGAGCGGAAGAACGGCGTAC          1     0
ATCGGAAGAGCGGTTCAGCAGGAATGCCGAGATCGGAAGATCGGTTCAT          1     0
```

In the above sample output, the first column contains the data from the second line of each fastq read, followed by the number of bases trimmed at the 5' end and the 3' end in the second and third columns respectively.

2.4.3 Fixed Trim (i.e., trimming off fixed number bases)

An alternative to BRAT's dynamic trimming function is a standard fixed trimming option we've included in the pipeline. While utilizing this option, the user has direct control over how many bases to trim from the 5' end and the 3' end by setting the options "-N" and "-n" respectively in the command line. The input and the output for the fixed trim option are the same as the input and output for BRAT large, so see section 2.4.1 for more information. Please note that it is good to use this trimming option if the users know exactly how many bases they want to trim off and no adaptor sequences have been trimmed yet. However, if the users have already trimmed the adapter sequences, then the reads length varies, it is NOT good to use this trimming option any more.

2.5 Step 3: BRAT Alignment and ACGT count

2.5.1 BRAT Large

BRAT Large handles the alignment of the data. The input is the output from Step 2, and the output will be labeled *.brat, a sample of which is shown below in box 6. For more information see the BRAT user manual, section BRAT large.

Box 6

```
trim.single.1M.brat
1     CGGGGAGTTAGCGTGAGAGGGGGGTTGGGTTAGTTAGTGTTCTTTTTTT    chr2 – 3296772   1 3296771
9     TGGGAAATTATAATGAGATTTGGTTTTCGAGAGTATT                chr2 + 74594669 0 74594669
13    TGGGTTTAAGTAATTTTTTTGTTTTAGTTTTTTAAGTAGTTGAGATC      chr2 + 45050947 0 45050947
```

2.5.2 ACGT-count

The input for this part of the pipeline is a bit unusual. Even though it utilizes the output from BRAT large, the input that is supposed to be included in the execution is actually a one-line file containing the name of the output from BRAT large. The output of ACGT-count is illustrated in box 2 of section 2.2.2.

2.6 Step 4.1: Data Parser

The remaining steps were developed by our research team.

2.6.1 Data Parser Input

The input for the data parsing step includes the BRAT ACGT-count initial input file and the target region input file that are explained in sections 2.2.2 and 2.2.3.

2.6.2 Data Parser Description

The first step for examining bisulfite rates using nonCGc sites is one of the most time-consuming portions of the pipeline. The main goal of this step is to prepare the large data files to be read into R-software so that the analysis output files are produced. If one were to examine the script executed in this section, the user would note that this parsing stage is completing many various tasks. First and foremost, the script separates positions belonging to an individual chromosome from the rest of the dataset. Then the script matches every base pair position to the corresponding target region (if a target file is provided). Next, the CG-type is examined for each site and CGc types are filtered from the data until only nonCGc sites remain. After a few minor adjustments, the data are now ready to be input into the final steps of the pipeline.

In section 2.7 and later, for “a good dataset”, we mean the results are from the quality assessment of MCF10A data; for “a bad dataset”, we mean the results are from the quality assessment of s7 data.

2.7 Step 4.2: Chromosome-level Analysis

2.7.1 Chromosome-level Analysis Input

The input for step 4.2 consists of the *.base files from Step 4.1 of the pipeline.

2.7.2 Chromosome-level Analysis Description

Step 4.2 is the first part of the analysis for the quality assessment of bisulfite-treated reads. Its purpose is to read each chromosome and produce a summary table so that the user can analyze each chromosome separately. It also produces a histogram to examine the bisulfite conversion rate for a specific chromosome specified by the user. At this point, the pipeline only deals with bisulfite-treated reads at the chromosome level. Analysis at the target region level will be described in Step 4.3.

2.7.3 Chromosome-level Analysis Output

Sample summary tables for “good” and “bad” datasets are shown in box 7.

Box 7

chr1.summary.table.txt (for the good dataset)

chr	TNCGC	TNCGCwC	percent	Min.	X1st.Qu.	Median	Mean	X3rd.Qu.	Max.
chr1	44683043	622926	0.01394	0	1	1	0.9961	1	1

chrX.summary.table.txt (for the bad dataset)

chr	TNCGC	TNCGCwC	percent	Min.	X1st.Qu.	Median	Mean	X3rd.Qu.	Max.
chrX	28558180	54784	0.0019	0	0.4725	1	0.7431	1	1

The summary table is structured to include chromosome number, total nonCGc sites, total nonCGc sites with coverage, and the percentage of nonCGc sites with coverage compared to the total number of nonCGc sites. The last 6 columns display a summary of the bisulfite rate for the entire chromosome including the minimum value, the 25% quartile, the median, average, the 75% quartile, and the maximum value. For the good dataset, there are 44683043 nonCGc sites in chr1 and 622926, or 1.394%, of those sites with coverage; for the bad dataset, there are 28558180 nonCGc sites in chr1 and 54784, or 0.2%, of those sites with coverage. This summary table shows the bisulfite conversion rate is very high in that the 25th percentile and mean are 1 and 0.996 respectively. The bottom chrX summary table in Box 7 is for a dataset with low bisulfite conversion rate in that the 25th percentile and mean are 0.47 and 0.74 respectively.

Figure 1 is the histogram of bisulfite rates from chromosome 1 in a "good" dataset. In this instance, an analysis of chromosome 1 would produce a histogram named prefix.chr1.BS.jpg in the output directory, which is illustrated in Figure 1 (also illustrated in Figure 1A in the main manuscript).

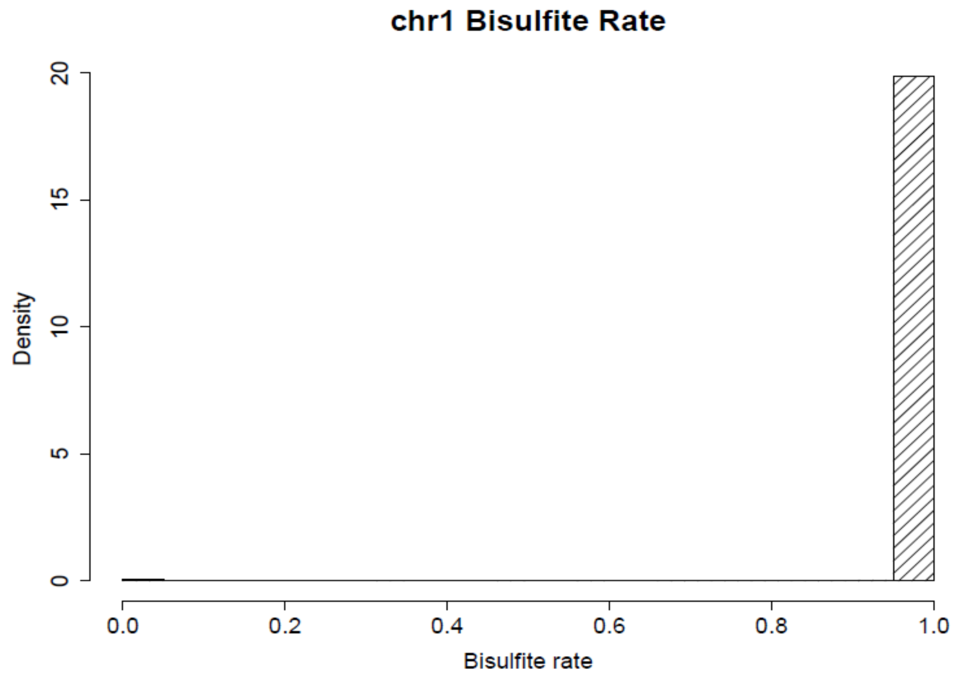

Figure 1: Histogram of bisulfite rates from chromosome 1 in a "good" dataset (i.e., a dataset with complete bisulfite conversion).

Figure 1 indicates that the bisulfite rate is very high and there is no evidence of incomplete bisulfite conversion. If a sequencing dataset has very low bisulfite conversion rates, the histogram will be very different from the plot shown in Figure 1. With low bisulfite conversion rates, the user will observe data points much less than one (see Figure 2). Figure 2 is a representative of a dataset with low bisulfite conversion rates, and many of the sites have a zero value implying that they are not converted at all.

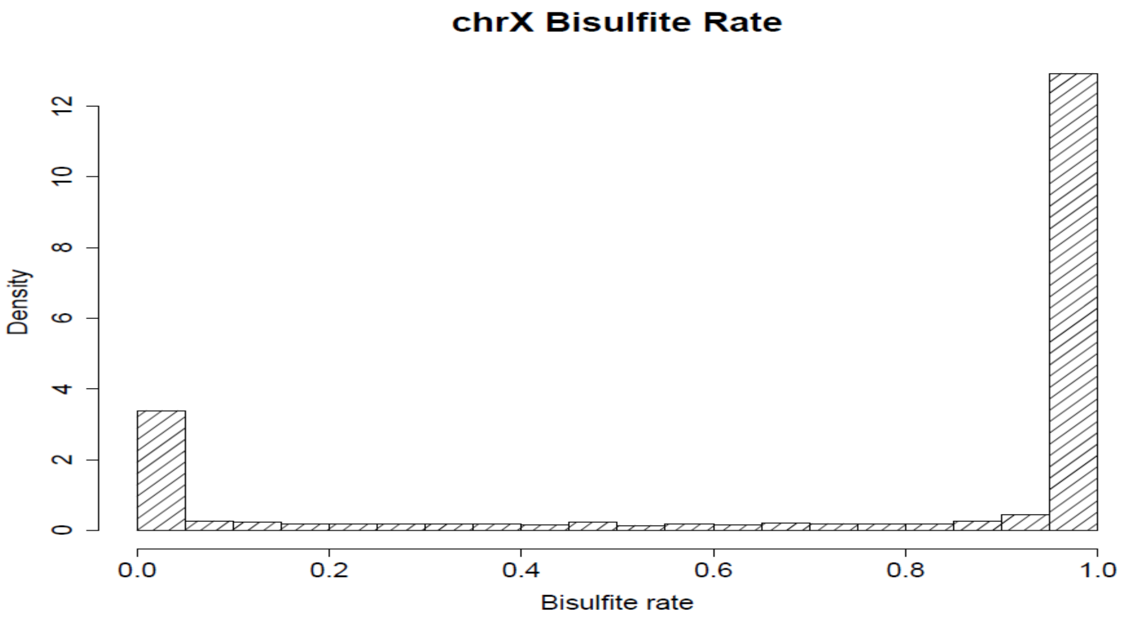

Figure 2: Histogram of bisulfite rates of chromosome 1 from a "bad" dataset (i.e., a dataset with incomplete conversion).

2.8 Step 4.3: Target-region Analysis

2.8.1 Target-region Analysis Input

The input for step 4.3 includes the *.nonCG.covered.out file from part 2 and the *.nonCG.match file from step 1.

2.8.2 Target-region Analysis Description

Step 4.3 of the pipeline begins the analysis at the target-region level. Its purpose is to read data from each target region and then produce a summary table and histogram to summarize the bisulfite-rates from each region.

2.8.3 Target-region Analysis Output

Step 4.3 of the pipeline analyzes all of the target regions for each chromosome and outputs a target summary table and a mean/median histogram. A sample of the target summary table is illustrated in box 8.

Box 8

chr1.target.summary.table.txt (for the good dataset)

chr	start	end	NCGC	NCGCwC	percent	Min.	X1st.Qu.	Median	Mean	X3rd.Qu.	Max.
chr1	100005824	100006023	27	3	0.1111	1	1	1	1	1	1
chr1	100026183	100026258	16	1	0.0625	1	1	1	1	1	1

chrX.target.summary.table.txt (for the bad dataset)

chr	start	end	NCGC	NCGCwC	percent	Min.	X1st.Qu.	Median	Mean	X3rd.Qu.	Max.
chrX	100694449	100694704	45	11	0.2444	0.9565	0.9891	1	0.9921	1	1
chrX	101657993	101658329	99	18	0.1818	0	0	0.01106	0.1745	0.0321	1

The above summary table is similar to the one from step 4.2 (i.e., chromosome level summary tables in Box 7). However, this summary table allows the user to analyze each target region individually. In Box 8, for the bad dataset, we see that some target regions have very low bisulfite rate summary results.

Figure 3 and 4 illustrate two examples of the figure (*.mean.median.ps file) that will be generated at the target-region level using the above summary table. They plot the mean and median bisulfite rates for all target regions on a specific chromosome. In particular, Figure 3 represents a set of data with high bisulfite conversion rate (i.e., bisulfite rate almost = 1); Figure 4 represents a set of data with low bisulfite rate, such that many target regions have mean or median bisulfite rates much lower than 1.

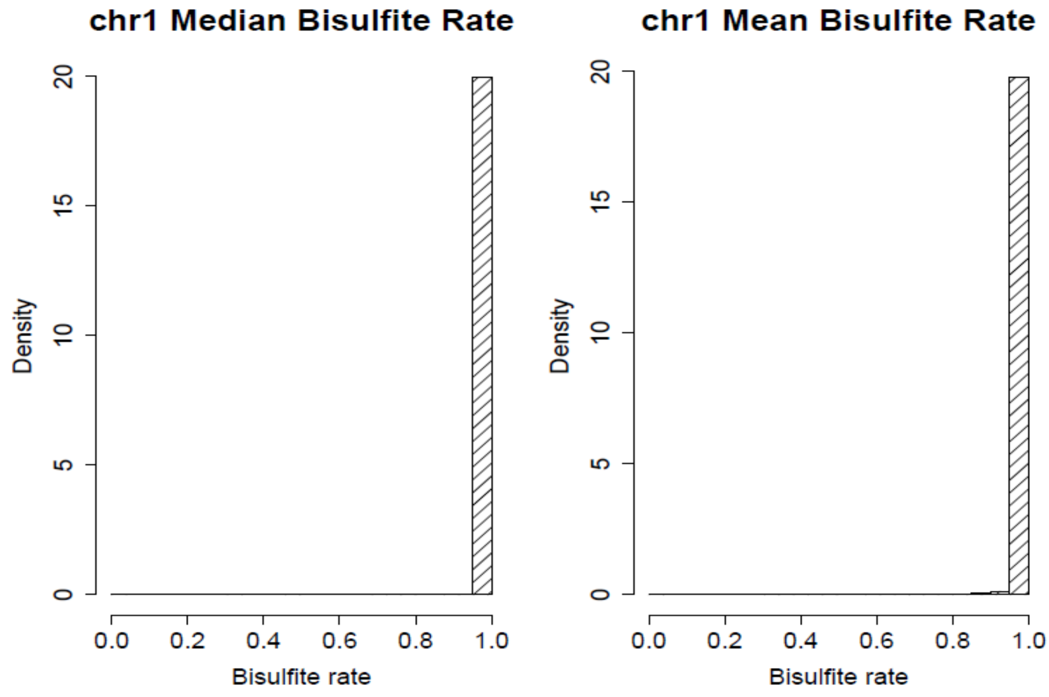

Figure 3: Histogram of mean and median bisulfite rates for target regions in chromosome 1. This figure represents mean and median bisulfite rates in a “good” dataset and is associated with the data from Figure 1. (Note: the target regions are RRBS intervals, not CpG islands.)

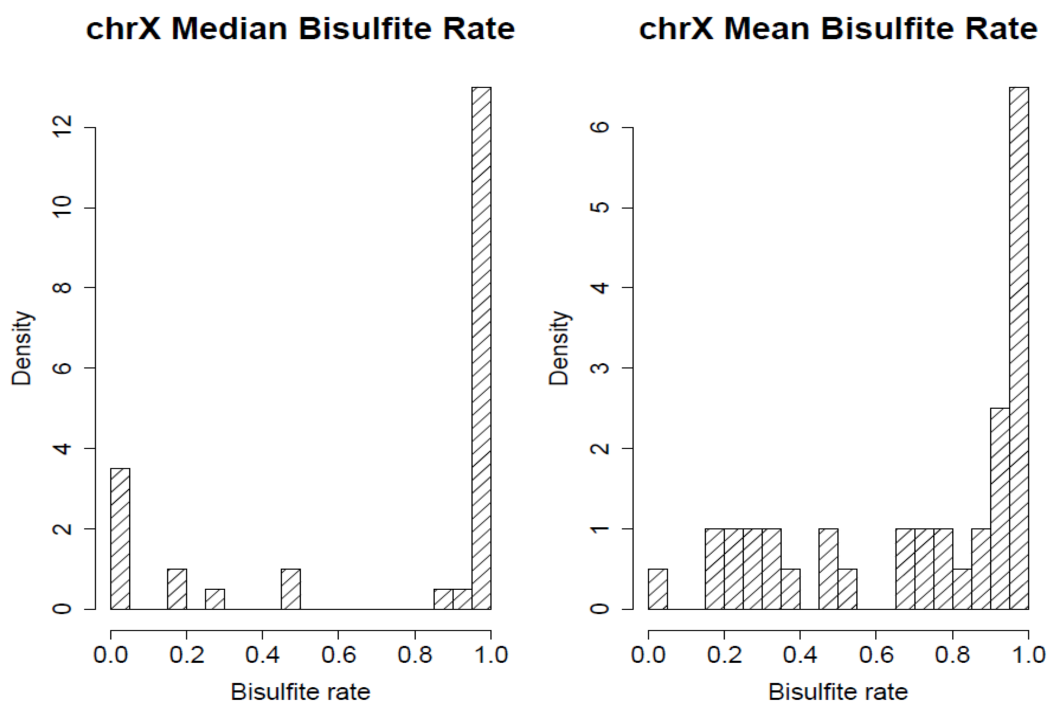

Figure 4: Histogram of mean bisulfite rates from a "bad" dataset, the same dataset employed in Figure 2. (Note: the target regions are CpG islands.)

2.9 Step 5: Sequence Structure Analysis

2.9.1 Sequence Structure Analysis Input

The input for step 5 is the target summary table from step 4.3.

2.9.2 Sequence Structure Analysis Description

Step 5 examines the reference sequence structure for regions with high or low bisulfite conversion rate and for regions with high or low coverage to provide insight for experimental scientists. The code in this step of the pipeline separates lines of the target summary table from step 4.3 according to high and low values set by the user for bisulfite rate and the coverage. For example, the user may specify to study the average reference structure of regions with bisulfite conversion rate higher than 90% and lower than 20% or any value desired in the command line. The same principle can also be applied to coverage of the reference structure of particular regions.

2.9.3 Sequence Structure Analysis Output

The output from step 5 will consist of a boxplot (e.g., Figure 5 and 6) analyzing the percentage of A, C, G, T, C+G, CGc, nonCGc and the percentage of repetitive bases for regions with high or low coverage or bisulfite conversion rate.

Figure 5 is for a good dataset that has no obvious sequencing problems, so there are no obvious DNS sequence structure differences. Figure 6 is for a bad dataset that has obvious bisulfite sequencing problems, so there are dramatic DNA sequence structure differences.

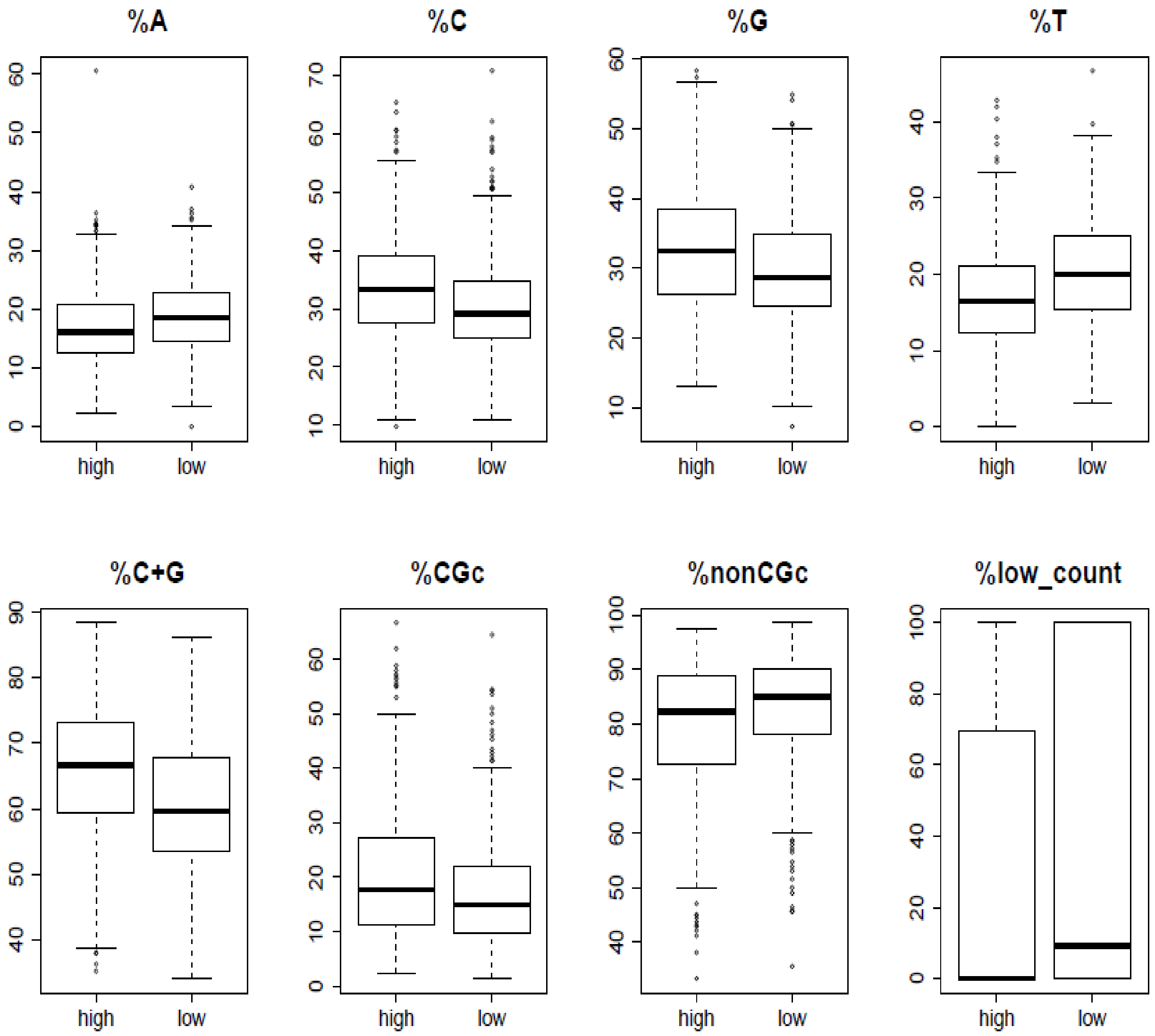

Figure 5: Boxplots comparing regions with high vs. low coverage in chromosome 1 from the "good" data, the same dataset employed in Figure 1. (Note: target regions are RRBS intervals.)

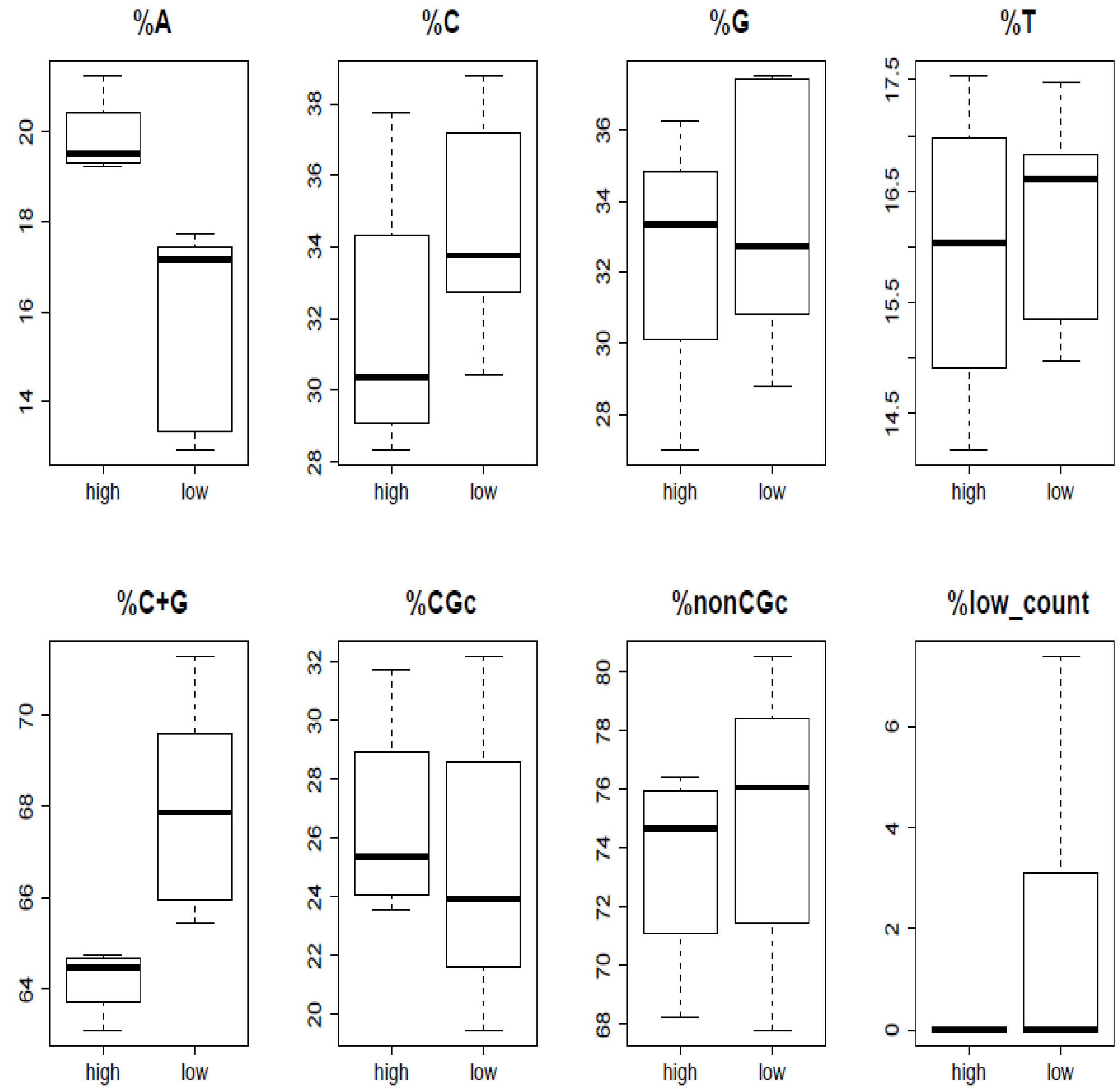

Figure 6: Boxplots comparing regions with high vs. low coverage in chromosome X from the "bad" data, the same dataset employed in Figure 2. (Note: target regions are CpG islands.)

Because the "bad" dataset we used has the incomplete bisulfite conversion problem, we also compared the DNA sequence structures for high and low bisulfite conversion regions (see Figure 7). Figure 7 shows that high bisulfite conversion regions tend to have relatively higher percentages of G and GC, but lower percentages of C and nonCGc. This comparison results show that regions with more Cs and nonCGcs tend to have incomplete bisulfite conversion problems in this sequencing experiment.

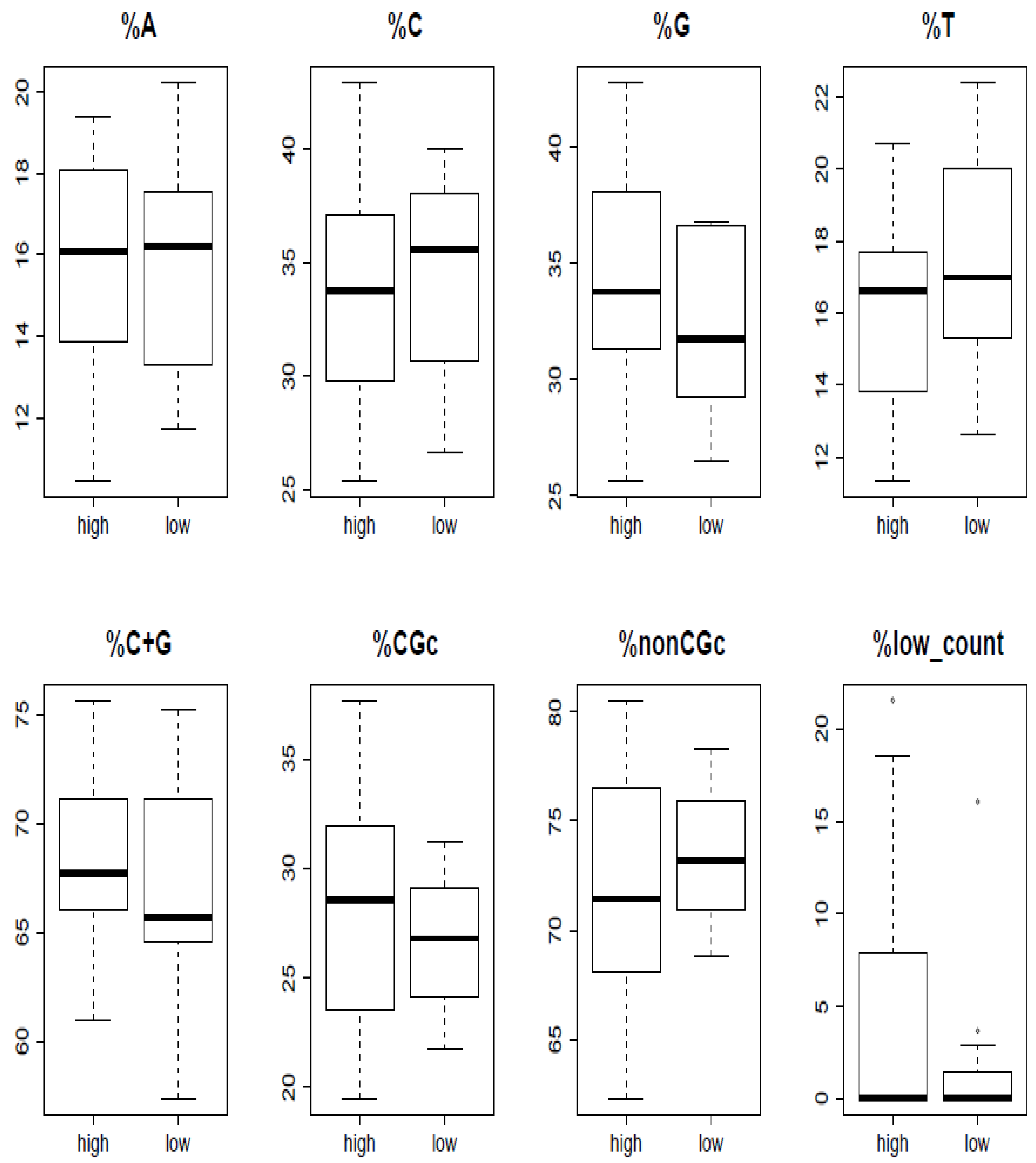

Figure 7: Boxplots comparing regions with high vs. low bisulfite rates in chromosome X from the "bad" data, the same dataset employed in Figures 2, 4 and 6. (Note: target regions are CpG islands.)

3. Additional Notes

3.1 System Requirements

To run the MethyQA pipeline, the user will need the following software:

- A Unix or Linux environment
- Installed Perl on system – in many cases Perl comes pre-installed in Linux and Mac systems, enter "perl –v" to see what version you have (if you do not have Perl, then you can download the latest version here: http://www.perl.org/get.html)
- Installed R on system – you can download the R software environment here: http://www.r-project.org/
- FastQC software – located here: http://www.bioinformatics.bbsrc.ac.uk/projects/fastqc/
- BRAT software – located here: http://compbio.cs.ucr.edu/brat/
- The user will also need a reference genome to do alignment.

3.2 Running Time

The main challenge of methylation-sequencing quality assessment is to process large amounts of data in a reasonably short amount of time. Current test-runs on our pipeline have produced a run-time of less than one hour from initiation of Step 1 through the completion of Step 5 for 20 million reads. These test-runs were done in a Red-hat Linux server with dual quad-core 2.66 GHz Xeon E5430 CPUs and 4 GB of RAM. An important note, however, is that the completion time of Step 3 (BRAT alignment and ACGT count) also depends on the number of reads for sites in the reference genome. Additionally, the run-time of the partial pipeline (Steps 4 and 5) is generally about 20 minutes.

3.3 Example

We have provided a README file (with the file name "README.txt") that can be downloaded from **http://hal.case.edu/~sun/MethyQA.v2.zip**. In the end of this file, we have included the script of running both the complete and partial MethyQA pipeline and the screen output of running these pipelines. In addition, in this README file, we have also given the explanation of the code and input folder.

3.4 Warning and trouble shooting notes

(1). If only the usage and command options are printed out when users are running the pipeline (methyQA.pl or partial.methyQA.pl), this problem may be due to one of the following reasons: (a) not all required options (-i, -t, -d, -c, -p, -R, -r) are provided; (b) even though they are provided, they are not provided correctly; (c) perl, R, and/or python are set as earlier versions. Thus, we recommend that users first check required options and the versions of perl, R and python carefully.

(2). If users get the following error messages,

(a) Error message:
"Traceback (most recent call last):

```
File "/meta/users/sun/May30.MethyQA.v3//resources/cutadapt/bin/cutadapt", line 9, in ?
  from cutadapt.scripts import cutadapt
File      "/meta/users/sun/May30.MethyQA.v3/resources/cutadapt/cutadapt/scripts/cutadapt.py",
line 71
  from .. import seqio, __version__
       ^
SyntaxError: invalid syntax"
```

(b) “ERRPR: if -a is set as cutadapt, -Y <path_python> has to be specified in command line"

For (a), it is very likely that the python version is not set up properly. For (b), the python version is not set up yet. When using cutadapt to do adapter trimming, "python2.6" (or python version 2.6) is required, changing the "-Y" option to be "-Y python2.6" will fix the problem.

(3). It is not suitable to set "-a cutadapt" and "-T fix" at the same time. This is because it is not meaningful to trim off reads with a fixed number of bases after adapter trimming.

(4). As for the plots of comparing DNA sequencing structures, we recommend that the user check the number of target regions in each of those high and low coverage (or bisulfite conversion) region files (using the “wc –l” unix/linux command). If there is a very small number (e.g., less than 10) of target regions in the low bisulfite or coverage region files, then the users may not worry about their data. For example, the following is the line-count for each respective sequencing file. It shows that there are only 6 target regions with relatively low bisulfite conversion rates.

```
5298   /meta/users/sun/May30.2013.test.partial.1/5.30testP.chr22.highBS.seq
3835   /meta/users/sun/May30.2013.test.partial.1/5.30testP.chr22.highCoverage.seq
6      /meta/users/sun/May30.2013.test.partial.1/5.30testP.chr22.lowBS.seq
82     /meta/users/sun/May30.2013.test.partial.1/5.30testP.chr22.lowCoverage.seq
```

4. References for software packages

[1] Andrews, S. (2010) FastQC: http://www.bioinformatics.bbsrc.ac.uk/projects/fastqc/.
[2] Harris, E.Y., *et al.* (2010) BRAT: bisulfite-treated reads analysis tool, *Bioinformatics*, **26**, 572-573.
[3] Marcel Martin (2010) Cutadapt: code.google.com/p/cutadapt/.
[4] Hannon, G.J. (2009) FASTX-Toolkit: http://hannonlab.cshl.edu/fastx_toolkit/.
